# A model for COVID-19 transmission in Connecticut

**DOI:** 10.1101/2020.06.12.20126391

**Published:** 2020-06-16

**Authors:** Olga Morozova, Zehang Richard Li, Forrest W. Crawford

**Affiliations:** 1 Department of Biostatistics, Yale School of Public Health; 2 Department of Statistics & Data Science, Yale University; 3 Department of Ecology & Evolutionary Biology, Yale University; 4 Yale School of Management

**Keywords:** SARS-CoV-2, mathematical modeling, SEIR model, lockdown, social distancing

## Abstract

To support public health policymakers in Connecticut as they begin phased lifting of social distancing restrictions, we developed a county-structured compartmental SEIR-type model of SARS-CoV-2 transmission and COVID-19 disease progression. We calibrated this model to the local dynamics of deaths and hospitalizations and the exact timing of state interventions, including school closures and stay-at-home order. In this technical report, we describe the details of the model design, implementation and calibration, and show projections of epidemic development through the Summer of 2020 under different assumptions about the increase in contact rates following partial state reopening. Our model results are consistent with high effectiveness of state lockdown measures, but changes in human interaction patterns during the coming months are unknown. In addition, a lot of uncertainty remains with respect to several key epidemiological parameters and the effectiveness of increased testing and contact tracing capacity. As more information becomes available, we will update the projections presented in this report.

## 1 Introduction

Epidemiological models of disease transmission play an important role in supporting public health decision-making. Trajectories from these models can provide insights into historical trends in epidemic dynamics or future outcomes under hypothetical intervention scenarios. Transmission models are especially useful in situations of high uncertainty, offering a structured way to assess the potential effects of interventions given plausible assumptions about disease transmission. Models cannot predict the future with certainty, but they can be helpful for scenario analysis by bounding the range of plausible future trajectories [1].

In the absence of effective pharmaceutical interventions, many countries, including the US, implemented social distancing measures and stay-at-home orders to slow transmission of SARS-CoV-2. As many states, including Connecticut, begin phased lifting of social distancing restrictions, public officials and public health policymakers are faced with several questions: 1) How effective are public health interventions like school closures and stay-at-home orders in reducing cases, hospitalizations, and deaths? 2) How should public health interventions be implemented in the future to minimize the risk of a resurgence? 3) What will be the effect of phased reopening plans? Multiple transmission models have been developed recently to help answer these and other policy questions [2–23]. When models are developed with a primary goal to support decision-making, they must balance parsimony and realism.

Projection models should be simple enough to fit to data, and should provide clear outputs in a timely manner with assumptions that can be understood by policymakers. At the same time, for such models to be useful, they need to be flexible enough to accommodate realistic epidemiological features and likely policy scenarios identified by stakeholders.

Many of the nation-wide COVID-19 forecasting models that have been developed in the United States either do not use local data, or make simplifying assumptions not appropriate for localities [2]. These models may capture national-level dynamics, but are less useful for supporting decision-making in individual states or counties. Several nation-wide models have been developed with a goal to provide projections at the state level [24–34]. These models employ varying methods, make different structural and parameter value assumptions, and project potential effects of different future policy interventions. They also vary in terms of model outputs and the ways of handling uncertainty. Most of these models use the same set of assumptions and estimates of key model parameters across all US states, and may not be able to capture important local variation.

In this technical report, we introduce a county-structured transmission model of SARS-CoV-2 transmission and COVID-19 disease progression in Connecticut. The model was developed with a goal to support intervention planning and decision-making in Connecticut, but could be adapted to other states or regions. This report provides an in-depth technical description of the model and data calibration approach, along with projections for Connecticut through the Summer of 2020. Additional COVID-19 reports for Connecticut in this series are available from https://crawford-lab.github.io/covid19_ct/.

## 2 Model specification

We developed a deterministic compartmental model of SARS-CoV-2 transmission and COVID-19 disease progression. The model is based on the SEIR (Susceptible, Exposed, Infectious, Recovered) framework [35], which we extend to accommodate geographical variation in Connecticut and distinct features of COVID-19 disease. We calibrate the model to observed dynamics of deaths and hospitalizations in Connecticut, and produce estimates of incidence and prevalence that may be helpful in designing population-level surveys. Similar models have been published recently, offering intervention effect estimates and projections in various locations [2, 3, 6, 7, 9, 13, 14, 36, 37].

**Figure 1:**
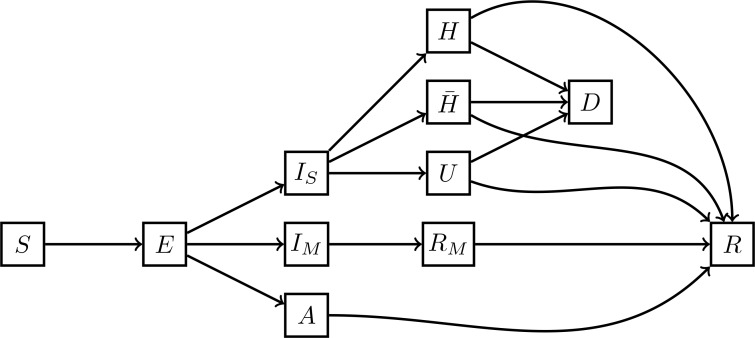
Schematic illustration of the SARS-CoV-2 transmission model and COVID-19 disease progression structure. Individuals begin in the susceptible (*S*) compartment. Exposed individuals (*E*) may develop either asymptomatic (*A*), mild (*I*_*M*_), or severe (*I*_*S*_) infection. Asymptomatic and mild infections resolve without hospitalization and do not lead to death. Mild symptomatic cases self-isolate (*R*_*M*_) shortly after development of symptoms, and transition to recovery (*R*) when infectiousness ceases. A proportion of severe cases require hospitalization (*H*) unless hospitalization capacity is exhausted, in which case they transition to $\bar{H}$ representing hospital overflow, then to recovery (*R*) or death (*D*). Some severe cases, including nursing homes and assisted living facilities residents, people in prisons, or individuals, who do not have access to hospitalization for other reasons, transition to compartment *U* and may later recover or die. The model assumes a closed population without births and does not capture non-COVID-19 deaths.

Figure 1 shows a schematic representation of the model structure. A mathematical description of the model, which includes information about how the model accommodates geographic variation across Connecticut counties, appears below. We categorize infections as asymptomatic, mild symptomatic, and severe. Only severe infections may lead to death. Severe infections are defined as those requiring hospitalization. Since one of the key observable features of the model is dynamics of hospitalizations in the community, we use separate compartments for community hospitalizations and for severe cases occurring in institutional settings, such as nursing homes, assisted living facilities, or prisons. The case fatality ratio (CFR) among severe cases in closed communities is assumed to be higher than the community hospital CFR. If hospitalization capacity is overwhelmed, some severe cases in the community are denied hospitalization, and experience a higher probability of death compared to hospitalized cases. Mild symptomatic infections are assumed to self-isolate shortly after they develop symptoms and remain isolated until they recover. During the infectiousness period of symptomatic cases, we assume a period of presymptomatic viral shedding, i.e. the latency period is shorter than incubation period [38–40]. In line with other similar models, we assume that individuals with asymptomatic infection exert a lower force of infection, but remain infectious for a longer period of time, since they are less likely to self-isolate in the absence of widespread testing [4, 9, 37]. The average time that severe cases spend in the infectious state is approximated by the time between onset of infectiousness and hospitalization (or attempted hospitalization in case of hospital overflow). Since severe cases are likely to be isolated at home before hospitalization, and those in nursing homes, assisted living facilities, or prisons are likely to be isolated within a few days after symptom onset, we assume reduced force of infection from severe infections in this setting. The force of infection from hospitalized patients to unhospitalized susceptible individuals is assumed to be negligible. We further assume that recovered individuals remain immune to reinfection for the duration of the study period. The model is implemented at the level of individual counties in Connecticut assuming that most contacts are happening within a given county. A small proportion of contacts is allowed to happen between adjacent counties. The analysis was performed using the R statistical computing environment [41]. We used package deSolve to perform numerical integration of the system of ordinary differential equations (ODE) [42].

### 2.1 Compartmental model and parameters

The model divides the population into 11 compartments: susceptible (*S*), exposed, latent infections (*E*), infectious and asymptomatic (*A*), infectious and mild symptomatic (*I*_*M*_), infectious and severe (*I*_*S*_), isolated mild infections removed from the pool of infectious individuals (*R*_*M*_), hospitalized (*H*), severe in need of hospitalization, but denied it due to hospital capacity overflow $\left(\bar{H}\right)$, severe in nursing homes, assisted living facilities, prisons or otherwise not having access to hospitalization (*U*), recovered (*R*), and died (*D*). Let *N*_*i*_ be the population size of county *i* and *J*_*i*_ the set of counties adjacent to county *i*. Let *C*^(*i*)^ represent hospitalization capacity in county *i*, which may vary over time. Transmission dynamics for county *i* are given by the following ODE system:

$$\frac{\text{d}S^{\left(i\right)}}{\text{d}t}=-\beta S^{\left(i\right)}\left[\left(1-k_{n}\right)\frac{I_{M}^{\left(i\right)}+k_{I_{S}}I_{S}^{\left(i\right)}+k_{A}A^{\left(i\right)}}{N_{i}}+\frac{k_{n}}{\left|J_{i}\right|}\sum_{j\in J_{i}}\frac{I_{M}^{\left(j\right)}+k_{I_{S}}I_{S}^{\left(j\right)}+k_{A}A^{\left(j\right)}}{N_{j}}\right],$$

$$\frac{\text{d}E^{\left(i\right)}}{\text{d}t}=\beta S^{\left(i\right)}\left[\left(1-k_{n}\right)\frac{I_{M}^{\left(i\right)}+k_{I_{S}}I_{S}^{\left(i\right)}+k_{A}A^{\left(i\right)}}{N_{i}}+\frac{k_{n}}{\left|J_{i}\right|}\sum_{j\in J_{i}}\frac{I_{M}^{\left(j\right)}+k_{I_{S}}I_{S}^{\left(j\right)}+k_{A}A^{\left(j\right)}}{N_{j}}\right]-\delta E^{\left(i\right)}$$

$$\frac{\text{d}A^{\left(i\right)}}{\text{d}t}=q_{A}\delta E^{\left(i\right)}-\alpha_{A}A^{\left(i\right)}$$

$$\frac{\text{d}I_{M}^{\left(i\right)}}{\text{d}t}=q_{I_{M}}\delta E^{\left(i\right)}-\alpha_{I_{M}}I_{M}^{\left(i\right)}$$

$$\frac{\text{d}R_{M}^{\left(i\right)}}{\text{d}t}=\alpha_{I_{M}}I_{M}^{\left(i\right)}-\gamma_{R_{M}}R_{M}^{\left(i\right)}$$

$$\frac{\text{d}I_{S}^{\left(i\right)}}{\text{d}t}=q_{I_{S}}\delta E^{\left(i\right)}-\alpha_{I_{S}}I_{S}^{\left(i\right)}$$

$$\frac{\text{d}H^{\left(i\right)}}{\text{d}t}=q_{H}\left(1-\rho^{\left(i\right)}\right)\alpha_{I_{S}}I_{S}^{\left(i\right)}-\gamma_{H}H^{\left(i\right)}$$

$$\frac{\text{d}{\bar{H}}^{\left(i\right)}}{\text{d}t}=q_{H}\rho^{\left(i\right)}\alpha_{I_{S}}I_{S}^{\left(i\right)}-\gamma_{\bar{H}}{\bar{H}}^{\left(i\right)},$$

$$\frac{\text{d}U^{\left(i\right)}}{\text{d}t}=\left(1-q_{H}\right)\alpha_{I_{S}}I_{S}^{\left(i\right)}-\gamma_{U}U^{\left(i\right)}$$

$$\frac{\text{d}D^{\left(i\right)}}{\text{d}t}=\gamma_{H}m_{H}H^{\left(i\right)}+\gamma_{\bar{H}}m_{\bar{H}}{\bar{H}}^{\left(i\right)}+\gamma_{U}m_{U}U^{\left(i\right)}$$

$$\frac{\text{d}R}{\text{d}t}=\alpha_{A}A^{\left(i\right)}+\gamma_{R_{M}}R_{M}^{\left(i\right)}+\gamma_{H}\left(1-m_{H}\right)H^{\left(i\right)}+\gamma_{\bar{H}}\left(1-m_{\bar{H}}\right){\bar{H}}^{\left(i\right)}+\gamma_{U}\left(1-m_{U}\right)U^{\left(i\right)}$$

The function *ρ*^(*i*)^ = [1 + exp(0.5(*C*^(*i*)^ − *H*^(*i*)^))]^−1^ is a “soft” hospitalization capacity overflow function.

Table 1 shows model parameters and their definitions. Some of the parameters from Table 1 are not directly used in the ODE system, but are used as inputs to compute other model parameters.

**Figure 2:**
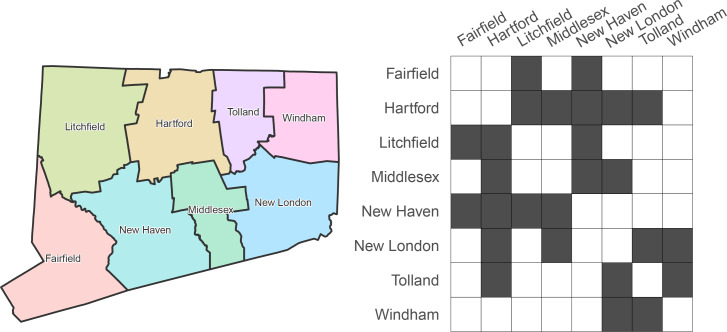
County map of Connecticut and county adjacency matrix. The dark gray cells correspond to counties that are adjacent. Contacts between adjacent counties are included in the model in addition to contacts within counties.

Figure 2 shows the county map of Connecticut along with the county adjacency matrix. The geographic boundary files were obtained from the Connecticut Department of Environmental Protection [43]. We assume that a fraction (1 − *k*_*n*_) of all contacts happen within a given county, and the remaining *k*_*n*_ contacts happen between individuals residing in adjacent counties.

### 2.2 Effects of social distancing and testing interventions

Social distancing measures, in particular school closure and lockdown, reduce the value of transmission parameter *β*. The value of the transmission parameter at time *t* is calculated as:

$$\beta (t)=\beta_{0}\left[1-\left(\iota_{\text{school}}(t)+\iota_{\text{lockdown}}(t)\right)\right],$$

where *β*_0_ is a value of transmission parameters in the absence of any interventions, *ι*_school_(*t*) is the proportion of contact reduction following school closure, and *ι*_lockdown_(*t*) is a proportion of contact reduction following lockdown that is independent of school closure effect. *ι*_school_(*t*) and *ι*_lockdown_(*t*) are zero when these interventions are not in effect, and can either have constant or time-varying values when interventions are in effect, subject to a constraint 0 ≤ *ι*_school_(*t*) + *ι*_lockdown_(*t*) ≤ 1 at any time *t*.

Based on the known dates of school closure and lockdown orders, and assuming that these effects are constant over time once they reach their maximum levels *w*_school_ and *w*_lockdown_, we calibrate the values of these effects to observed data. To project epidemic dynamics into the future, we replace *ι*_lockdown_(*t*) with a step function representing post-lockdown increases in population-level contact. Figures 5 to 10 show examples of assumed post-lockdown increases in contact, starting on May 20, 2020.

Increase in testing capacity and contact-tracing is assumed to result in earlier identification and isolation of mild symptomatic and asymptomatic cases, and leads to increases in *α*_*A*_ (rate of transition from *A* to *R*) and $\alpha_{I_{M}}$ (rate of transition from *I*_*M*_ to *R*_*M*_). The values of these transition rates at time *t* are calculated as:

$$\;\alpha_{A}(t)=\alpha_{A}^{\left(0\right)}\left[1+\iota_{\text{testing},A}(t)\right]\;\alpha_{I_{M}}(t)=\alpha_{I_{M}}^{\left(0\right)}\left[1+\iota_{\text{testing},I_{M}}(t)\right],$$

where $\alpha_{A}^{\left(0\right)}$ and $\alpha_{I_{M}}^{\left(0\right)}$ are reciprocals of the duration of time until isolation among asymptomatic and mildly symptomatic infections respectively under current testing availability and contact tracing conditions. Similarly to the parametrization of distancing effects, *ι*_testing, *A*_(*t*) and $\iota_{\text{testing},I_{M}}(t)$ may vary over time. We assume that soon after the lockdown is lifted, testing capacity and contact tracing efforts increase and remain constant at that level. The values of *ι*_testing, *A*_(*t*) and $\iota_{\text{testing},I_{M}}(t)$ are assumed to be zero prior to May 20, 2020 and increase to *w*_testing, *A*_ and $w_{\text{testing},I_{M}}$ respectively afterwards.

## 3 Model calibration and Bayesian posterior inference

We calibrate the distribution of model parameters using the observed dynamics of community hospitalizations and cumulative number of deaths. Due to changing testing patterns and varying case ascertainment proportions over time, we do not use reported case counts in the calibration procedure [1, 44]. As is often the case for epidemic models, not all parameters are simultaneously identifiable given the observed data on hospitalizations and deaths. In particular, different sets of parameters that collectively determine early epidemic growth rate (i.e. transmission parameter *β* and parameters that define force of infection) are observationally equivalent. Given this limitation, we fix a subset of parameters at their point estimates and estimate the remaining parameters using a Bayesian approach. To generate uncertainty intervals for projections, we sample from the joint posterior over estimated parameters and uncertainty distributions for a subset of fixed parameters, then find pointwise 95% posterior predictive intervals for each time point.

Several model parameters influence epidemic dynamics in the future, beyond the data available for calibration. These parameters include the hospital overflow CFR $\left(m_{\bar{H}}\right)$, testing capacity and contact tracing effects (*w*_testing, *A*_ and $w_{\text{testing},I_{M}}$), and the extent of release of suppressed contact once lockdown is lifted. These parameters cannot be estimated based on historical data, but may be estimated in the future. For the purpose of projections, we impose a distribution on $m_{\bar{H}}$, *w*_testing, *A*_, and $w_{\text{testing},I_{M}}$ independent of one another and of the joint posterior distribution of calibrated parameters. Increases in contact rates following state reopening are parameterized relative to the size of lockdown effect *w*_lockdown_. The point estimates of models parameters, prior distributions, and assumed distributions are summarized in Table 2.

### 3.1 Continuous transmission model parameters

Let *θ* denote the subset of continuous transmission model parameters whose joint distribution is calibrated to observed data. For each individual parameter *θ*, we specify a fixed support [*θ*_min_*, θ*_max_], and put independent beta priors on the transformed parameter, i.e.,

(1) $$\frac{\theta -\theta_{\min}}{\theta_{\max}-\theta_{\min}}∼\mathrm{Beta}\left(a_{\theta },b_{\theta }\right),$$

where the shape parameters *a*_*θ*_ and *b*_*θ*_ are set to let *θ* have mean *μ*_*θ*_ and standard deviation *σ*_*θ*_. Table 2 provides the list of (*μ*_*θ*_*, σ*_*θ*_*, θ*_min_*, θ*max) for all parameters in *θ*.

### 3.2 Initial conditions

Given a set of transmission parameters, we initialize the compartmental model by specifying the size of exposed (*E*) compartment in each county and setting the size of downstream compartments to be zero. Let $E_{0}^{\left(i\right)}$ be the *i*-th element of vector *E*_0_ denoting initial size of exposed compartment in county *i*. The initial size of susceptible compartment in county *i* is given by $S_{0}^{\left(i\right)}=N_{i}-E_{0}^{\left(i\right)}$. Elements of vector *E*_0_ were pre-specified based on the relative population size and the dates of first registered case and death in each county.

We allow the date of epidemic onset to vary by letting *E*_0_ to correspond to day −*L*_0_, where day 0 corresponds to a calendar date of March 1, 2020. The state of the system at any given time is therefore a deterministic function of the initial size of exposed compartment, model parameters, and *L*_0_ that determines the date of epidemic onset. We put a uniform prior on *L*_0_ over {*l*_min_*, …, l*_max_}.

### 3.3 Reported hospitalizations and deaths

We accommodate reporting lags in observed hospitalizations and deaths. Reporting lags are correlated with other unknown parameters, including latency period, epidemic onset lag, time between infection and hospitalization, time between infection and death, length of hospital stay, and difference in death reporting lags between hospitals and nursing homes. Following Osthus et al. [45], we model the observed population fractions of hospitalizations *h*(*t*) and deaths *d*(*t*) at time *t* using a Beta distribution,

(2) $$h(t)∼\mathrm{Beta}\left(\lambda_{h}\frac{H\left(t,L_{0},L_{H},\theta \right)}{N},\;\lambda_{h}\left(1-\frac{H\left(t,L_{0},L_{H},\theta \right)}{N}\right)\right),$$

(3) $$d(t)∼\mathrm{Beta}\left(\lambda_{d}\frac{D\left(t,L_{0},L_{D},\theta \right)}{N},\;\lambda_{d}\left(1-\frac{D\left(t,L_{0},L_{D},\theta \right)}{N}\right)\right),$$

where *N* is the size of Connecticut population; and *H*(*t, L*_0_*, L*_*H*_*, θ*) and *D*(*t, L*_0_*, L*_*D*_*, θ*) are model-projected current hospitalizations and cumulative deaths at time *t* with reporting lags *L*_*H*_ and *L*_*D*_, and epidemic onset lag *L*_0_ under parameter values *θ*. We put uniform priors on *L*_*H*_ and *L*_*D*_ over a range of plausible values. Parameters *λ*_*h*_ and *λ*_*d*_ control the variance of respective Beta distributions. We put the same independent Gamma prior on *λ*_*h*_ and *λ*_*d*_, i.e.

(4) $$\lambda ∼\mathrm{Gamma}\left(s_{\lambda },r_{\lambda }\right),$$

where *s*_*λ*_ and *r*_*λ*_ are shape and rate parameters of the Gamma distribution, set to *s*_*λ*_ = 4 and *r*_*λ*_ = 0.0002.

### 3.4 Posterior inference

**Figure 3:**
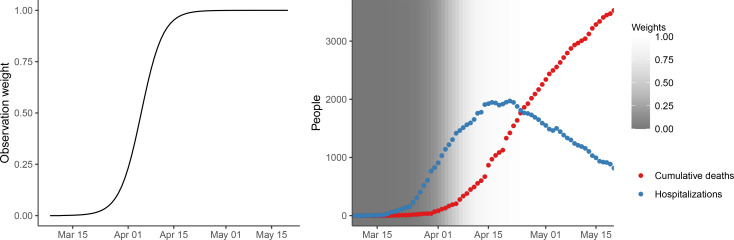
Weight function used in the weighted likelihood. The assigned weights are close to 1 after April 12.

Posterior inference is performed using a weighted likelihood that assigns more weight to more recent observations. We let the weight function *z*(*t*) take the following form,

(5) $$z(t)=\frac{1}{1+\exp\left(-k_{z}t\right)},$$

where *z*(*t*) is the weight assigned to observation at time *t*, *t* ∈ {*t*_0_*, …,t*_max_} and the correspondence between {*t*_0_*, …,t*_max_} and calendar time is set such that *t* = 0 corresponds to *M* = 45 days prior to the most recent observation. Parameter *k*_*z*_ controls the smoothness of logistic function. We set *k*_*z*_ = 0.3, which leads to the weight function in Figure 3.

We construct the posterior distribution over unknown parameters (***θ, λ,* L**) as

(6) $$p(\theta ,\lambda ,L|h(t),d(t))\propto p(\theta )p(\lambda )p(L)\prod_{t}{\left[p\left(h(t)|H\left(t,L_{0},L_{H},\theta \right),\lambda_{h}\right)p\left(d(t)|D\left(t,L_{0},L_{D},\theta \right),\lambda_{d}\right)\right]}^{z(t)/2},$$

where $\theta =\left(q_{I_{S}},\beta ,\gamma_{H},\gamma_{U},k_{A},k_{I_{S}},m_{H},m_{U},q_{HD},w_{\text{school}},w_{\text{lockdown}}\right)$, ***λ*** = (*λ*_*h*_*,λ*_*d*_), and **L** = (*L*_0_, *L*_*H*_*, L*_*D*_). Each likelihood term is weighted by *z*(*t*)/2 because we assign equal weight 1/2 to data contributions from both hospitalizations and deaths. Sampling from the joint posterior distribution of (***θ, λ***, **L**) given in (6) is performed using Markov Chain Monte Carlo (MCMC). We implemented a Metropolis-Hastings algorithm with random walk proposals for ***θ*** and ***λ*** and independent proposals for **L**. We ran the sampler for 2,000,000 iterations and thinned the chain using every 400th iteration.

### 3.5 Parameter values, prior distributions, and data sources

Asymptomatic infections play an important role in transmission of SARS-CoV-2 [38–40, 46], but estimates of the proportion of infections that do not exhibit symptoms vary [46–51]. The true proportion of asymptomatic infections is important for projections and policy planning due to its relationship to evolving herd immunity. In the absence of reliable estimates of cumulative incidence based on sero-prevalence surveys, we consider three scenarios:

1. Scenario 1, low asymptomatic: *q*_*A*_ = 0.36
2. Scenario 2, medium asymptomatic: *q*_*A*_ = 0.5
3. Scenario 3, high asymptomatic: *q*_*A*_ = 0.7

The value *q*_*A*_ = 0.36 was calculated as an age-adjusted weighted average of several estimates available in the literature. Nishiura et al. [48] estimated 30.8% among Japanese citizens evacuated from Wuhan, China. We applied this estimate to age group 20–64 years old. Mizumoto et al. [49] estimated 17.9% among infections on the Diamond Princess cruise ship. We applied this estimate to age group 65 plus years old, which is the age group, in which most infections occurred. We assumed 60% for age group 0–19 years old, consistent with findings reported by Russell et al. [52], where 4 out of 6 infections in this age group were asymptomatic among passengers of the Diamond Princess. The average was weighted by the age distribution of Connecticut population. At the time of writing this report, the best estimate of asymptomatic proportion recommended by the CDC was 35% [53]. Based on recent evidence suggesting that the proportion of asymptomatic infections may be higher [46, 47, 50, 51, 54, 55], we assumed *q*_*A*_ = 0.5 and *q*_*A*_ = 0.7 in medium and high scenarios respectively.

Table 2 provides a list of prior mean, standard deviation, and lower and upper bounds of the model parameters along with data sources. For parameters whose values were fixed, only the mean is given. In the three scenarios considered, we fixed the values of asymptomatic proportions (*q*_*A*_). The proportion of severe cases $\left(q_{I_{S}}\right)$ was allowed to vary with a mean assumed to be 10% of symptomatic infections in line with [14, 56–58].

Duration of the latency period (1/*δ*) was fixed, since reliable estimates of this parameter are available and because it is correlated with parameters that were allowed to vary, including reporting lags and parameters that determine duration of infectiousness and time between infection and recovery or death. We assume an average of 4 days of latency [14, 36] and 1.5 days of presymptomatic infectiousness [59] resulting in an average incubation period of 5.5 days in line with [14, 47, 53, 57, 60–62].

Parameters (*α*_*A*_, *k*_*A*_, $\alpha_{I_{M}}$, $\alpha_{I_{S}}$, $k_{I_{S}}$) collectively determine force of infection at a given time. Force of infection and transmission parameter *β* together determine the early growth of the epidemic. Without additional data, these parameters cannot be simultaneously identified. We therefore fixed parameters for which estimates are available or which can be calculated from available estimates (*α*_*A*_, $\alpha_{I_{M}}$, $\alpha_{I_{S}}$), and assumed prior distributions and calibrated posterior of those that are unavailable (*k*_*A*_, $k_{I_{S}}$). Duration of infectiousness of asymptomatic individuals is unknown, but is likely shorter than that of symptomatic individuals [63]. While several studies estimated that viral RNA could be detected in upper respiratory tract for 2–3 weeks [64, 65], findings from [66] suggest that live virus can be isolated for a substantially shorter time period, which in this study was a maximum of 7 days. We assume a diffuse prior on *β*, which absorbs additional variations of parameters that determine force of infection.

#### Initial conditions and reporting lags

Based on the relative population size of the counties and the date of first registered case and death, we assume an initial size of exposed compartment to be 7.5 in Fairfield, 6.5 in Hartford, 0 in Litchfield, 0.25 in Middlesex, 4.5 in New Haven, 0.13 in New London, 0.05 in Tolland and 0.04 in Windham. We allow the starting date of the epidemic to be between February 12th – 19th and calibrate a scenario-specific distribution of starting dates to the observed data.

Early in the epidemic, many infections were likely “imported” from New York City, which experienced a major outbreak in the spring of 2020. In particular, many residents of Fairfield county work in New York City. We do not directly model importation events: the assumed number of exposed individuals at time zero in Fairfield county does not include all such events. Infection importation from New York City likely continued for days or even weeks early in the epidemic. This unaccounted force of infection is absorbed in the estimation of transmission parameter *β*.

We specified a wide range of reporting lags for hospitalizations and deaths, resulting in ranges of 5–9 days for hospitalizations and 8–11 days for deaths. It is likely that deaths occurring in hospitals are reported with substantially smaller lag than those occurring in nursing homes, since many of the latter ones need to be confirmed by a medical examiner [77].

#### Reduction in transmission following school closure and lockdown orders

We calibrated the parameters that determine reduction in contact rates following school closure and lockdown orders to the observed data. We assume that *ι*_school_(*t*) equals zero before March 13th, 2020 - the date of school closure order, and equals some constant positive value *w*_school_ afterwards. The *ι*_lockdown_(*t*) equals zero before March 20th, 2020 - the date of lockdown order, and equals some constant positive value *w*_lockdown_ after March 23rd, 2020 - the date when the lockdown order took effect. Between March 20th - March 23rd, 2020 the value of *ι*_lockdown_(*t*) is smoothly increasing from zero to *w*_lockdown_. Posterior parameter estimates were similar if we allowed a smoother decrease in contact rates. Since the dates of school closure and lockdown orders are very close, it is difficult to simultaneously identify *w*_school_ and *w*_lockdown_. We therefore put a narrow prior distribution on *w*_school_ and diffuse on *w*_lockdown_, subject to a constraint 0 ≤ (*w*_school_ + *w*_lockdown_) ≤ 1.

#### Observed data

Cumulative deaths and current hospitalizations were obtained from Connecticut Department of Public Health daily reports [76] and the Connecticut Hospital Association [75]. We calculated county-level population and age structure in Connecticut using the 2014–2018 estimates of the American Community Survey [78]. This analysis does not use individual-level patient data.

Daily total available hospital beds (including occupied) in each county were obtained from the Connecticut Hospital Association/CHIMEData [75, 79] and used as hospitalization capacity values on a given date. The latest available value of hospitalization capacity was used for future projections.

## 4 Results

**Figure 4:**
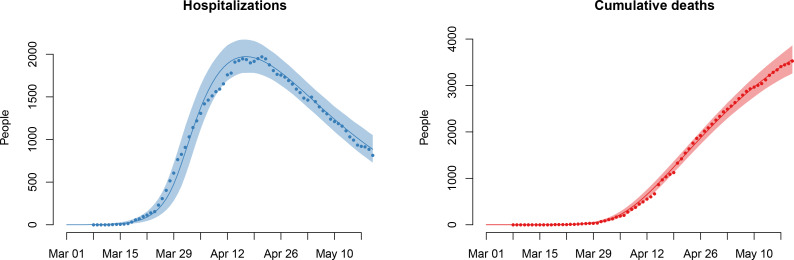
Parameter calibration results under the medium asymptomatic fraction scenario (*q*_*A*_ = 0.5).

Figure 4 shows projections of hospitalizations and deaths, with 95% posterior predictive intervals, under the medium asymptomatic fraction scenario and observed data as dots. Observed data points track with mean projections and largely fall within uncertainty intervals. Calibration results under low and high asymptomatic fraction scenarios demonstrate a similar fit to historical data. Table 3 reports means and 95% credible intervals of marginal posterior distributions of model parameters and derived epidemiological parameters under the three asymptomatic fraction scenarios.

**Figure 5:**
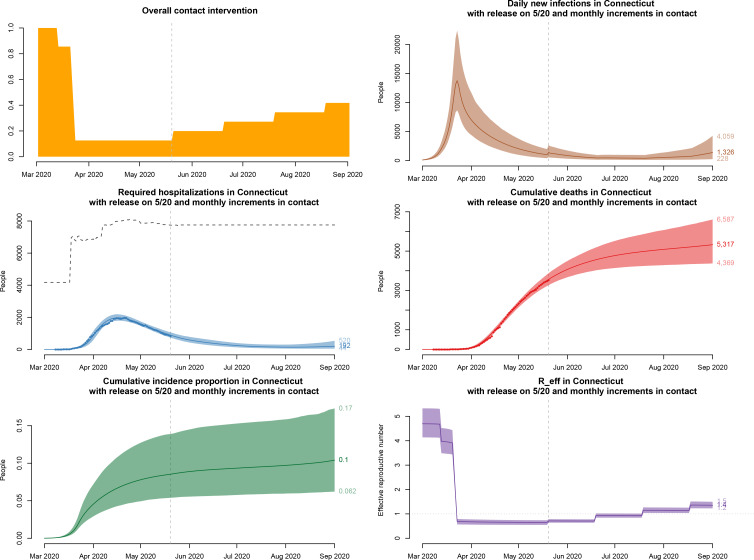
Projections under a “slow” reopening scenario and Scenario 1, low asymptomatic proportion. 10% of suppressed contact is released every 30 days, starting on May 20, 2020, with 95% uncertainty intervals. The dashed line above hospitalization projections is an estimate of the hospital bed capacity in Connecticut [75]. *R*_eff_ in March includes the effect of imported infections from outside Connecticut, in particular the New York region.

**Figure 6:**
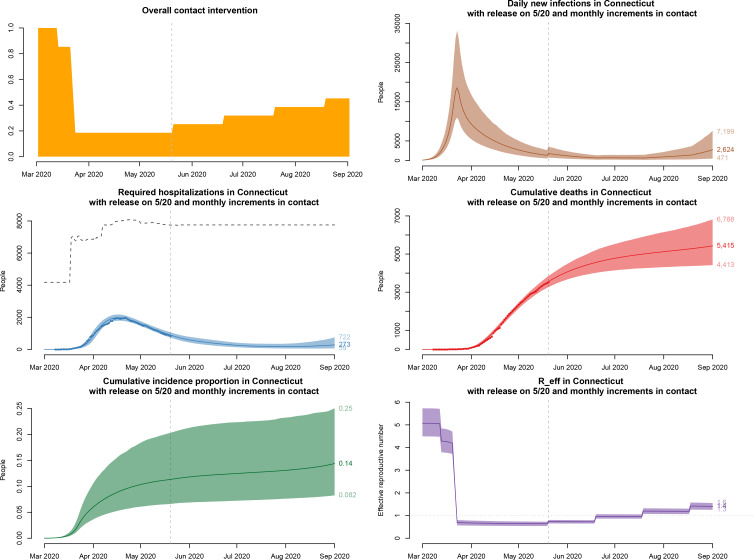
Projections under a “slow” reopening scenario and Scenario 2, medium asymptomatic proportion. 10% of suppressed contact is released every 30 days, starting on May 20, 2020, with 95% uncertainty intervals. The dashed line above hospitalization projections is an estimate of the hospital bed capacity in Connecticut [75]. *R*_eff_ in March includes the effect of imported infections from outside Connecticut, in particular the New York region.

**Figure 7:**
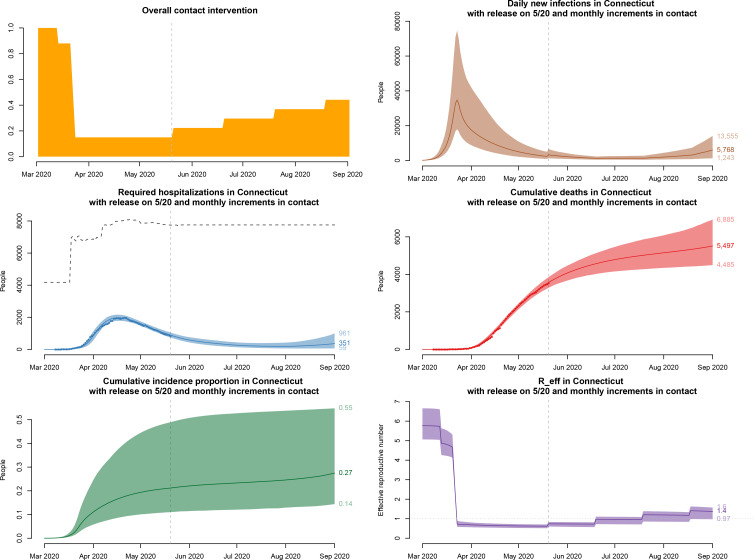
Projections under a “slow” reopening scenario and Scenario 3, high asymptomatic proportion. 10% of suppressed contact is released every 30 days, starting on May 20, 2020, with 95% uncertainty intervals. The dashed line above hospitalization projections is an estimate of the hospital bed capacity in Connecticut [75]. *R*_eff_ in March includes the effect of imported infections from outside Connecticut, in particular the New York region.

**Figure 8:**
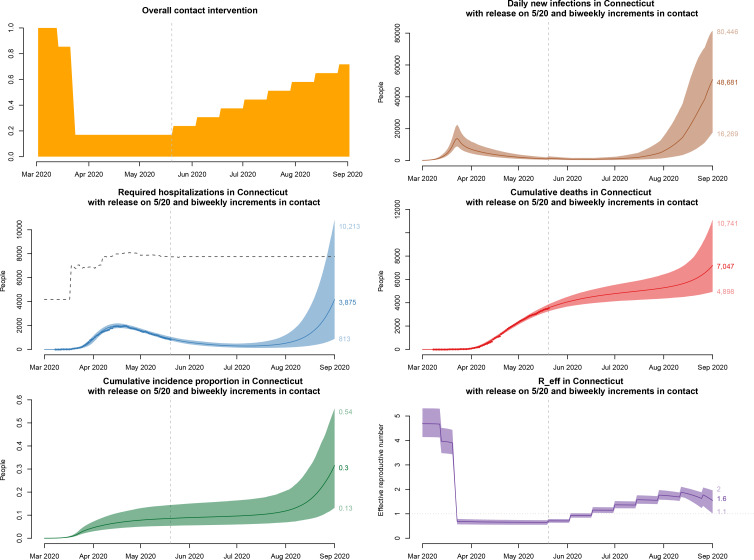
Projections under a “fast” reopening scenario and Scenario 1, low asymptomatic proportion. 10% of suppressed contact is released every 2 weeks starting on May 20, 2020, with 95% uncertainty intervals. The dashed line above hospitalization projections is an estimate of the hospital bed capacity in Connecticut [75]. *R*_eff_ in March includes the effect of imported infections from outside Connecticut, in particular the New York region.

**Figure 9:**
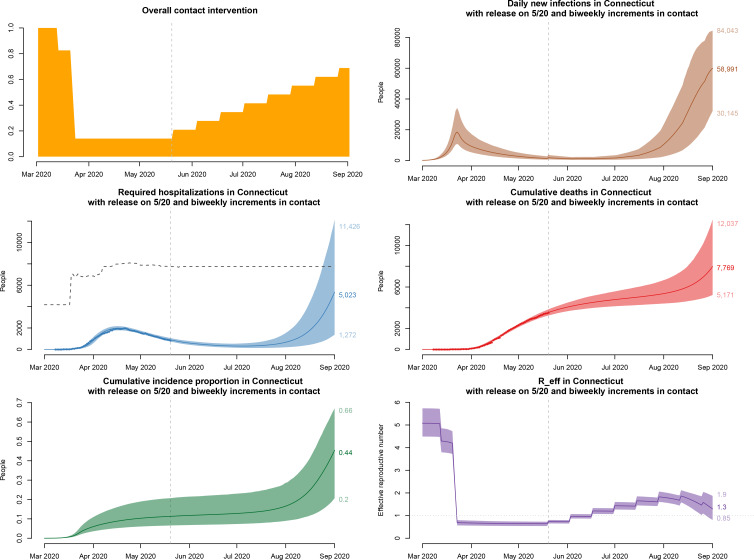
Projections under a “fast” reopening scenario and Scenario 2, medium asymptomatic proportion. 10% of suppressed contact is released every 2 weeks, starting on May 20, 2020, with 95% uncertainty intervals. The dashed line above hospitalization projections is an estimate of the hospital bed capacity in Connecticut [75]. *R*_eff_ in March includes the effect of imported infections from outside Connecticut, in particular the New York region.

**Figure 10:**
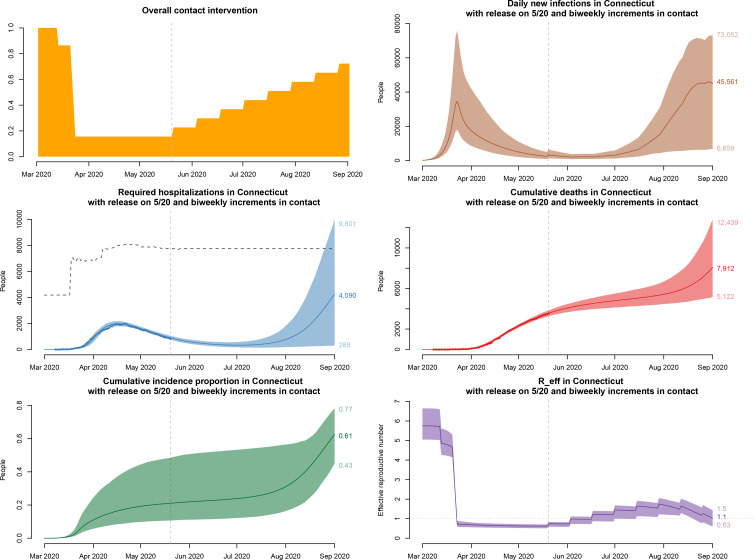
Projections under a “fast” reopening scenario and Scenario 3, high asymptomatic proportion. 10% of suppressed contact is released every 2 weeks, starting on May 20, 2020, with 95% uncertainty intervals. The dashed line above hospitalization projections is an estimate of the hospital bed capacity in Connecticut [75]. *R*_eff_ in March includes the effect of imported infections from outside Connecticut, in particular the New York region.

Figures 5 to 7 summarize projections under the low, medium and high asymptomatic fraction and ‘slow’ reopening. Slow reopening assumes that at one-month intervals, 10% of the contacts suppressed via lockdown is released. Figures 8 to 10 summarize projections for the three asymptomatic fraction scenarios assuming ‘fast’ reopening. Fast reopening assumes that 10% of the suppressed lockdown contact is released at two-week intervals. The projections include daily COVID-19 incidence, hospital census, deaths, cumulative incidence proportion, and effective reproductive number, *R*_eff_.

Our estimate of “crude” *R*_0_ under the low asymptomatic fraction scenario is 4.69 (95% CI: 4.14 − 5.33), which is on the higher end of available estimates [3, 4, 13, 36, 47, 53, 61, 80], but similar to recent modeling studies that estimated *R*_0_ to be between 4.7 and 6.3 in Northeastern United States, and around 5 in New York and New Jersey [7, 80]. Under the high asymptomatic fraction scenario, our estimate of crude *R*_0_ is 5.76 (95% CI: 5.05 − 6.65). We believe that this over-estimates the true *R*_0_ as we do not model infection importation events from New York City that likely made a substantial contribution to the force of infection early in the epidemic. Unaccounted force of infection biases estimates of transmission parameter *β*, and hence our “crude” *R*_0_, upwards. A recently published analysis implies that *R*_0_ = 3.3 in Connecticut [81]. This suggests that about 30% to 40% of infection events early in the epidemic could be imported, which is a plausible proportion range. Estimates of *R*_eff_ at later stages of the epidemic are less likely to be affected by this source of bias, since lockdown measures have probably resulted in a very low rate of interstate contacts. Our estimates of *R*_eff_ on May 20 are similar under the three asymptomatic proportion scenarios.

Under high asymptomatic fraction scenario, we would expect a higher number of infections, hospitalizations and deaths by the end of summer. Under slow reopening, hospitalization capacity is not expected to be overwhelmed under any of the scenarios. In case of fast reopening, hospitalization capacity would likely be exceeded sooner if asymptomatic fraction is high. The main difference between the asymptomatic fraction scenarios is in the cumulative incidence proportion. By the end of summer, it may be as low as 0.1 under the low asymptomatic fraction and slow reopening, and as high as 0.6 under the high asymptomatic fraction and fast reopening. The latter scenario predicts the second peak of infections to occur in mid-August, while all other scenarios predict that the second peak would not occur until later in the Fall. While higher asymptomatic fraction is expected to result in higher number of deaths in the short-term, the total number of deaths by the end of the epidemic would likely be higher under low asymptomatic fraction. When asymptomatic fraction is low, infections and deaths are delayed, but the proportion of severe cases is higher.

## 5 Discussion

In this report, we have described technical details of a model of SARS-CoV-2 transmission and COVID-19 disease progression developed to support public health decision-making in Connecticut. The model is calibrated to the observed dynamics of hospitalizations and cumulative deaths in Connecticut; its projections reproduce these dynamics accurately. Many COVID-19 models have been developed and analyzed by the CDC in the attempt to perform ensemble forecasting of the epidemic development in the US [2]. Some of these models offer state-level projections [24–34]. The CDC publishes updates of consolidated summary of cumulative death projections in the next four weeks from these models for each state. Local projections from nation-wide models may offer useful insights, however simplifying or uniform assumptions made in most of these models may not hold in all of the locations.

There is substantial uncertainty about epidemiological parameters that govern aspects of COVID-19 dynamics and have a direct impact on the quality of projections. When local context is not directly taken into account, the effects of parametric uncertainty is exacerbated. State and county-level models are needed to support local decision-making. Our model captures distinct important features of COVID-19 dynamics and the relationship between model features and data reporting in Connecticut. By calibrating model parameters to local data, including changes in hospitalization capacity, and the exact timing of intervention events, we can reduce uncertainty in projections. However, the calibrated posterior distribution of model parameters is not necessarily generalizable to other settings: model projections are tightly linked to the Connecticut reopening plans.

In addition to providing predictions for policymakers, model projections may be useful for prospectively planning epidemiological studies that can inform the state’s response. In particular, planning of seroprevalence surveys requires estimates of the proportion of population who have evidence of prior exposure to the virus. Due to limited testing availability and potentially high proportion of asymptomatic individuals, official case counts offer a poor approximation to the true cumulative incidence. Seroprevalence surveys, if properly conducted, can provide an important piece of information that would permit more precise estimates of the fraction of asymptomatic infections.

Our estimates of symptomatic case hospitalization ratio (CHR) and symptomatic CFR are higher than those recommended by the CDC for modeling studies [53]. Epidemic dynamics in Connecticut may be different from other states and from the average dynamics across the US. Our prior estimates are informed by the reports of Connecticut Department of Public Health [76, 77], as well as data provided by the Connecticut Hospital Association [75], and by Yale New Haven Hospital. To the extent possible, we use a data-driven approach to parameter estimation, and our posterior parameter estimates are highly consistent with observed epidemic dynamics. One of the important factors that may explain relatively high estimates of CFR in Connecticut is related to high mortality among residents of nursing homes and assisted living facilities. About 50% of official COVID-19 related deaths in Connecticut occurred in this subpopulation [77]. Lower CHR implies a lower proportion of severe cases, and would push estimates of CFR higher in order to reproduce the observed dynamics of hospitalizations and deaths simultaneously.

Symptomatic CHR, symptomatic CFR, and infection fatality ratio (IFR) are related epidemiologic quantities that describe severity of infection. Early estimates of these parameters used in many modeling studies come from China [56, 57, 82]. A recent review suggests that COVID-19 has higher severity in Europe and in the US compared to China [58] with higher CFR in the same age groups [83]. Estimates of CFR among hospitalized patients range from 15.4% to 29% in different parts of the US [58, 68, 71], and were reported to be as high as 32% - 38.6% in the UK [70, 74]. These differences suggest that some epidemiologic parameters may not be generalizable between different settings, and that, to the extent possible, local projections should rely on local parameter estimates.

Estimates of IFR that rely on official COVID-19 related death counts are likely biased downwards [84]. While a recent systematic review reports a pooled estimate of IFR = 0.75% [85], studies that attempt correction of this source of bias report IFR estimates of 1.04% [86] and 1.29% [87], consistent with our model estimates. Epidemiological parameters provided by the CDC are regularly updated and may change in the future.

Prior knowledge and assumptions about plausible ranges of parameter values combined with local data allows us to substantially reduce parametric uncertainty and produce narrow projection intervals. However, several important considerations limit our ability to make reliable long-term projections. First, it is difficult to make predictions about the extent of contact rate increases following lockdown release steps, such as allowing only certain types of businesses to reopen. Second, the effectiveness of widespread testing and contact tracing on timely isolation of infectious individuals, and its subsequent impact on the force of infection is unknown. This effect is a complex function of viral shedding characteristics among symptomatic, presymptomatic and asymptomatic individuals, along with the implementation features of contact tracing, testing, and isolation [88]. Multiple studies conclude that social distancing is highly effective in reducing transmission with effect estimates similar to our estimate of combined school closure and lockdown effect [6, 13, 14, 16, 18]. Connecticut officials plan increases in contact tracing and testing capacity to mitigate potential increases in contact rates, and several modeling studies suggest that combination of reduced social distancing with increased testing may prevent a second wave of infections [8, 9], but testing coverage should be very high and isolation should happen rapidly [13]. It is, however, unclear how feasible is repeated population-level screening and how successful is traditional “manual” contact tracing. Some researchers argue that while repeated testing is important for health-care workers and high risk groups, it is unlikely to have a substantial impact on transmission [89]. Contact tracing using mobile technology may be a promising intervention [17]. Overall, our estimates suggest that at the end of May, 2020 *R*_eff_ was around 0.65, meaning that a substantial level of social distancing would need to be maintained long-term and that testing and contact tracing alone would likely be insufficient to keep *R*_eff_ below 1.

Third, there may be unequal depletion of susceptible individuals depending on their severity risk profile. If a higher fraction of high-risk individuals have already experienced infection compared to low-risk individuals, then we may be under-estimating current prevalence and over-estimating future number of deaths and the final epidemic size [22, 23]. Case counts and mortality data from nursing homes suggest that susceptible individuals in this group are likely depleted faster than the in general population [77], but there may be other high risk groups, which have not yet experienced major outbreaks. While uniform reduction in contact rates following lockdown is consistent with observations, lower risk groups may have reduced their contacts slower than high-risk groups, resulting in higher cumulative incidence with the majority of infections being asymptomatic [51].

Fourth, we assume that all epidemiological parameters are constant over time and are not subject to seasonal forcing. It is hypothesized that temperature may play a role in transmission dynamics beyond its impact on contact patterns [3], in which case we would expect lower transmission intensity in summer all other things being equal. A recent study, however, suggests that the overall impart of warm temperature is small and insufficient to reduce transmission during summer months below the epidemic threshold in the absence of other interventions [90].

In an evolving public health crisis, it is important to keep projections up to date. As more information about the extent of asymptomatic transmission, general epidemiological characteristics of COVID-19, and data on observable model features in Connecticut becomes available, we will update the projections presented in this paper. Reports in this series are posted to https://crawford-lab.github.io/covid19_ct/.

## Figures and Tables

**Table 1:** Transmission model parameters.

Notation	Definition
*β*	Transmission parameter per *S* – *I* pair
*δ*	(Latency period)^−1^ (days^−1^)
*q_A_*, $q_{I_{M}}$, $q_{I_{S}}$	Proportions of asymptomatic, mild symptomatic, and severe infections, sum to one
*q_H_*	Proportion of severe cases that will result in hospitalization or attempted hospitalization under hospital overflow
*q_HD_*	Proportion of all COVID-19 related deaths that occur in hospitals
*α_A_*	(Duration of infectiousness among asymptomatic infections)^−1^ (days^−1^)
$\alpha_{I_{M}}$	(Duration of infectiousness among mild symptomatic infections, time until isolation)^−1^ (days^−1^)
$\gamma_{R_{M}}$	(Duration of isolation among mild symptomatic infections, time to recovery) ^−1^ (days^−1^)
$\alpha_{I_{S}}$	(Duration of infectiousness among severe cases, time until hospitalization)^−1^ (days^−1^)
*γ_H_*	(Length of hospitalization, time until recovery or death)^−1^ (days^−1^)
$\gamma_{\bar{H}}$	(Remaining time until recovery or death among hospital overflow patients)^−1^ (days^−1^)
*γ_U_*	(Remaining time until recovery or death among nursing home residents and other closed communities)^−1^ (days^−1^)
*k_A_*	Relative infectiousness of asymptomatic cases compared to symptomatic
$k_{I_{S}}$	Isolation coefficient among severe cases in nursing homes and those expecting hospitalization
*m_H_*	Case fatality ratio among hospitalized severe cases
$m_{\bar{H}}$	Case fatality ratio among severe cases denied hospitalization due to hospital capacity overflow
*m_U_*	Case fatality ratio among severe cases in nursing homes, prisons, or with no access to hospitalization
*k_n_*	Proportion of all contacts that happen with individuals from adjacent counties (as opposed to within county)
*C*(*t*)	Hospitalization capacity at time *t*, may be constant or vary over time representing capacity increase intervention
*w*_school_	Maximum size of school closure effect on contact rate reduction
*w*_lockdown_	Maximum size of lockdown effect on contact rate reduction
*w*_testing, *A*_	Effect size of increased testing and contact tracing on the asymptomatic isolation rate
$w_{\text{testing},I_{M}}$	Effect size of increased testing and contact tracing on the mildly symptomatic isolation rate
*L_H_*, *L_D_*	Reporting lags of hospitalizations and deaths (days)
*L*_0_	Lag of epidemic onset date, days before March 1, 2020

**Table 2:** Prior distributions of model parameters

Parameter	Mean	SD	Lower	Upper	Source
*q_A_*: low	0.36	-	-	-	[48, 49, 52]
*q_A_*: medium	0.5	-	-	-	Assumed, in line with [46, 47, 54]
*q_A_*: high	0.7	-	-	-	Assumed, in line with [46, 47, 54]
$q_{I_{S}}$ (low *q_A_*)	0.064	0.013	0.014	0.114	10% of symptomatic infections [14, 56–58]
$q_{I_{S}}$ (medium *q_A_*)	0.05	0.013	0.01	0.09	10% of symptomatic infections [14, 56, 57]
$q_{I_{S}}$ (high *q_A_*)	0.03	0.009	0.005	0.055	10% of symptomatic infections [14, 56, 57]
*β* (low *q_A_*)	1.25	0.5	0.25	2.25	A diffuse prior assumed
*β* (medium *q_A_*)	1.5	0.5	0.25	2.75	A diffuse prior assumed
*β* (high *q_A_*)	2	0.5	0.5	3.5	A diffuse prior assumed
*δ*	1/4	-	-	-	[14, 47, 57, 59–62]
*α_A_*	1/7	-	-	-	[63, 66, 67]
$\alpha_{I_{M}}$	1/4	-	-	-	[3, 14, 36]; of 4 days, 1.5 is assumed to be presymptomatic [59]
$\gamma_{R_{M}}$	1/7	-	-	-	[66, 67]
$\alpha_{I_{S}}$	1/9.5	-	-	-	[56, 68–70]
*γ_H_*, $\gamma_{\bar{H}}$	1/10	0.015	0.04	0.16	[58, 68, 71, 72], Yale New Haven Hospital (personal communication)
*γ_U_*	1/14	0.01	0.03	0.11	Assumed
*k_A_*	0.4	0.05	0.2	0.6	[4, 37]
$k_{I_{S}}$	0.7	0.05	0.5	0.9	Assumed
*m_H_*	0.2	0.015	0.1	0.3	[56, 58, 68, 70, 71, 73–75]
*m_U_ / m_H_*	1.5	0.15	1	2	Assumed
$m_{\bar{H}}/m_{H}$	1.5	0.25	1	2	Assumed
$q_{HD}$	0.48	0.012	0.36	0.6	[75, 76]
*q_H_*	0.58	-	-	-	Calculated based *q_HD_* and *m_U_ / m_H_*
*k_n_*	0.015	-	-	-	Assumed
*w*_school_	0.15	0.015	0.1	0.2	Assumed
*w*_lockdown_	0.7	0.05	0.55	0.85	Assumed
*w*_testing, *A*_	0.2	0.03	0.1	0.3	Assumed
$w_{\text{testing},I_{M}}$	0.5	0.04	0.35	0.65	Assumed

**Table 3:** Means and 95% credible interval of marginal posterior distributions of model parameters and epidemiological parameters under the low (*q_A_* = 0.36), medium (*q_A_* = 0.5), and high (*q_A_* = 0.7) asymptomatic fraction scenarios.

Parameter	low asymptomatic			medium asymptomatic			high asymptomatic		
	mean	CI-low	CI-high	mean	CI-low	CI-high	mean	CI-low	CI-high
$q_{I_{S}}$	0.063	0.038	0.089	0.049	0.025	0.074	0.028	0.011	0.046
*β*	1.26	1.09	1.46	1.44	1.23	1.70	1.81	1.48	2.19
*γ_H_*	0.112	0.095	0.131	0.113	0.095	0.132	0.114	0.097	0.131
*γ_U_*	0.072	0.053	0.091	0.072	0.053	0.090	0.071	0.052	0.089
*k_A_*	0.40	0.30	0.49	0.40	0.30	0.49	0.39	0.30	0.49
$k_{I_{S}}$	0.70	0.60	0.80	0.70	0.60	0.80	0.70	0.60	0.80
*m_H_*	0.209	0.183	0.236	0.209	0.182	0.234	0.208	0.184	0.233
*m_U_*	0.314	0.248	0.386	0.313	0.246	0.390	0.311	0.243	0.386
*q_H_*	0.58	0.52	0.63	0.58	0.52	0.63	0.58	0.52	0.62
*w*_school_	0.15	0.12	0.18	0.15	0.12	0.18	0.15	0.12	0.18
*w*_lockdown_	0.70	0.65	0.74	0.71	0.67	0.75	0.71	0.67	0.75
crude *R*_0_	4.69	4.14	5.33	5.07	4.49	5.73	5.76	5.05	6.65
*R*_eff_ on May 20, 2020	0.65	0.55	0.74	0.64	0.54	0.73	0.62	0.51	0.71
symptomatic CHR	0.056	0.034	0.081	0.056	0.029	0.086	0.054	0.021	0.088
IHR	0.036	0.022	0.052	0.028	0.014	0.043	0.016	0.006	0.027
symptomatic CFR	0.025	0.014	0.036	0.025	0.013	0.038	0.023	0.009	0.039
IFR	0.0159	0.0091	0.0232	0.0123	0.0064	0.0189	0.0070	0.0026	0.0118

CHR, case hospitalization ratio (proportion of symptomatic cases requiring hospitalization); IHR, infection hospitalization ration (proportion of all infections requiring hospitalization); CFR, case fatality ratio (proportion of symptomatic cases who die); IFR, infection fatality ration (proportion of all infections who die).
