# Supplementary material for: One year of modeling and forecasting COVID-19 transmission to support policymakers in Connecticut
Source: medRxiv. 2021 Apr 23:2020.06.12.20126391. Originally published 2020 Jun 16. Preprint. [Version 3] doi: 10.1101/2020.06.12.20126391 (PMC7310630; doi:10.1101/2020.06.12.20126391)
Supplement: 1 [file NIHPP2020.06.12.20126391-supplement-1.pdf]

medRxiv preprint doi: https://doi.org/10.1101/2020.06.12.20126391; this version posted April 23, 2021. The copyright holder for this preprint (which was not certified by peer review) is the author/funder, who has granted medRxiv a license to display the preprint in perpetuity.
It is made available under a CC-BY 4.0 International license .

Supplementary Material: Detailed Methods

Estimating hospitalizations and hospital deaths coming from non-congregate settings

Our transmission model aims to capture disease spread in the community and excludes residents of congregate settings, whose contact patterns violate homogeneous mixing assumption. Available hospitalizations data do not disaggregate by the patient's place of residence at the time of diagnosis or hospitalization. We have therefore estimated the time-varying number of hospital admissions and hospitalizations census that came from congregate settings and excluded them from the observed time series. We received data on daily COVID-19 death counts in hospitals disaggregated by the type of residence (congregate vs. non-congregate) at the time of diagnosis or hospitalization from Connecticut Department of Public Health (CT DPH). Posterior distribution of model parameters was calibrated to the estimated time series of cumulative hospitalizations and hospitalizations census coming from non-congregate settings and to the observed time series of hospital deaths in this population.

The time-varying proportion of hospitalization census and cumulative hospitalizations coming from congregate settings was estimated as follows:

1. We first estimated the cumulative number of hospitalizations coming from congregate settings as the total number of hospital deaths among residents of congregate settings divided by the hospital case fatality ratio (HFR) among this population, and adjusting for an average hospital length of stay. The estimated HFR among residents of congregate settings in Connecticut is 0.38 and was obtained from the CT DPH based on a survey of a sample of 50 nursing homes, representing about a quarter of all nursing homes in Connecticut [29].

2. Next, we computed the cumulative proportion of hospitalizations coming from congregate settings as an estimate of the number of cumulative hospitalizations coming from congregate settings divided by the total cumulative number of hospitalizations as of the same date.

3. The proportion of hospital admissions coming from congregate settings varied over time. To approximate temporal dynamics of this proportion, we assumed that it follows the same pattern as the time-varying proportion of deaths among hospitalized residents of congregate settings among all hospital deaths:

$$p_{\text{hospital congregate deaths}}(t) = \frac{d_{\text{hospital congregate deaths}}(t)}{d_{\text{hospital deaths}}(t)},$$

where $d(t)$ denotes smoothed daily death counts calculated as a first order difference of spline-smoothed

medRxiv preprint doi: https://doi.org/10.1101/2020.06.12.20126391; this version posted April 23, 2021. The copyright holder for this preprint (which was not certified by peer review) is the author/funder, who has granted medRxiv a license to display the preprint in perpetuity.
It is made available under a CC-BY 4.0 International license .

cumulative counts of respective time series. We then re-scaled this time-varying proportion relative to the cumulative proportion of deaths among hospitalized residents of congregate settings among all hospital deaths and lagged it back by the average hospital length of stay. The resulting time-varying function was then multiplied by the cumulative proportion of hospitalizations coming from congregate settings estimated in step 2 to obtain a time-varying estimate of the proportion of hospital admissions coming from congregate settings at time t.

This time-varying proportion was then applied to the CHA-reported daily hospital census and daily hospital admissions to estimate the number of current hospitalizations, daily hospital admissions, and cumulative hospitalizations coming from congregate settings, and respective time series coming from non-congregate settings follow directly.

Data smoothing for time-varying parameters

All time-varying parameters described in detail in the Methods section rely on smoothed functions of observed data. Figure 5 shows five functions of observed data series overlaid by a smooth function (red line), which is used as a component of time-varying model parameters.

Model calibration and Bayesian posterior inference

We calibrate the posterior distribution of model parameters to the estimated hospitalizations and hospital deaths coming from non-congregate settings using a Bayesian approach with a Gaussian likelihood. Model-based estimates of observed quantities are adjusted for reporting lags. The distributions of hospitalizations census, cumulative hospitalizations, and hospital deaths are given by:

$$h(t) \sim \mathcal{N}\left(H(t, L_H, \boldsymbol{\theta}), \sigma_h^2\right), \tag{2}$$

$$u(t) \sim \mathcal{N}\left(U(t, L_H, \boldsymbol{\theta}), \sigma_u^2\right), \tag{3}$$

$$d(t) \sim \mathcal{N}\left(D(t, L_D, \boldsymbol{\theta}), \sigma_d^2\right), \tag{4}$$

where $H(t, L_H, \boldsymbol{\theta})$, $U(t, L_H, \boldsymbol{\theta})$, and $D(t, L_D, \boldsymbol{\theta})$ are model-projected hospitalizations census (lagged by L_H), cumulative hospitalizations (lagged by L_H), and cumulative deaths (lagged by L_D) at time t under parameter values $\boldsymbol{\theta}$. Prior distributions imposed on calibrated model parameters ensure non-negative values of model compartments.

medRxiv preprint doi: https://doi.org/10.1101/2020.06.12.20126391; this version posted April 23, 2021. The copyright holder for this preprint
(which was not certified by peer review) is the author/funder, who has granted medRxiv a license to display the preprint in perpetuity.
It is made available under a CC-BY 4.0 International license .

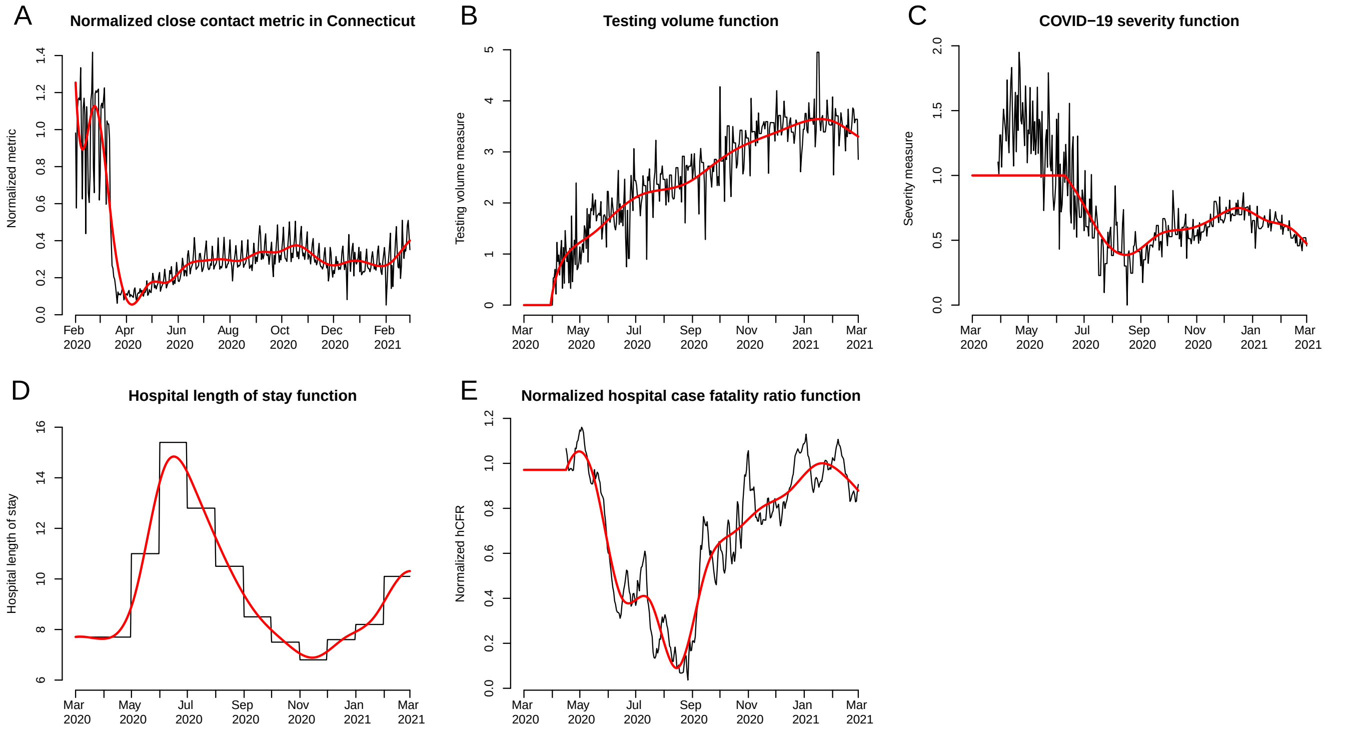

Figure 5: Smooth functions of observed data used as components of time-varying model parameters. A: normalized close contact metric, function $M_{\text{contact}}(t)$; B: testing volume function $M_{\text{testing}}(t)$; C: COVID-19 severity function, normalized proportion of cases 60+ years old among all cases detected at time t, function $M_{\text{severity}}(t)$; D: average hospital length of stay among COVID-19 patients, reciprocal of rate of hospital discharge $\gamma_H(t)$; E: normalized hospital case fatality ratio, function $M_{\text{HFR}}(t)$. Black lines show input data trajectories and red lines show smooth functions of observed data.

medRxiv preprint doi: https://doi.org/10.1101/2020.06.12.20126391; this version posted April 23, 2021. The copyright holder for this preprint (which was not certified by peer review) is the author/funder, who has granted medRxiv a license to display the preprint in perpetuity.
It is made available under a CC-BY 4.0 International license .

We put uniform priors on L_H and L_D over a range of plausible integer values. Reporting lags are correlated with other unknown parameters, including latency period, time between infection and hospitalization, time between infection and death, and length of hospital stay, therefore L_H and L_D should not be strictly interpreted as reporting lags. We put the same independent Inverse-Gamma(a, b) prior on all three hyperparameters σ_h^2, σ_u^2, and σ_d^2 with $a = 0.5$ and $b = 5 \times 10^6$ at the end of the modeling period. The prior was gradually relaxed as observed time series increased. For the purposes of future forecasting, as opposed to estimation of epidemiologic features from the past model dynamics, we imposed a tighter prior on these hyperparameters to achieve reasonable posterior predictive intervals beyond the observation period.

We construct the posterior distribution over unknown parameters $(\boldsymbol{\theta}, \boldsymbol{\sigma})$ as:

$$
\begin{aligned}
p(\boldsymbol{\theta}, \boldsymbol{\sigma} | h(t), u(t), d(t)) \propto p(\boldsymbol{\theta}) p(\boldsymbol{\sigma}) & \prod_{t \in t_H} \left[p\left(h(t) | H(t, L_H, \boldsymbol{\theta}), \sigma_h\right) \right]^{w_h z(t)} \\
& \prod_{t \in t_U} \left[p\left(u(t) | U(t, L_H, \boldsymbol{\theta}), \sigma_u\right) \right]^{w_u z(t)} \\
& \prod_{t \in t_D} \left[p\left(d(t) | D(t, L_D, \boldsymbol{\theta}), \sigma_d\right) \right]^{w_d z(t)},
\end{aligned} \tag{5}
$$

where $\boldsymbol{\theta} = (\beta_0, q_A, \alpha_{I_S}, m_{H,0}, E_0, L_H, L_D, \tau, \boldsymbol{\epsilon})$ and $\boldsymbol{\sigma} = (\sigma_h, \sigma_u, \sigma_d, \sigma_\epsilon)$.

We assume the date of epidemic onset to be February 16th, 2020 - 21 days before the first case was officially confirmed in Connecticut on March 8th, 2020, and initialize the model with E_0 exposed individuals at the time of epidemic onset, setting the size of all downstream compartments to be zero. County-level distribution of E_0 is fixed and was estimated based on the county population size and dates of first registered case and death in each county.

Each likelihood term is weighted by the time-dependent weight $z(t)$ times the weight assigned to a respective time series. We let the weight function $z(t)$ take the following form,

$$
z(t) = \frac{1}{1 + \exp(-k_z t)}, \tag{6}
$$

where $z(t)$ is the weight assigned to an observation at time t, $t \in \{t_0, ..., t_{\max}\}$, and the correspondence between $\{t_0, ..., t_{\max}\}$ and calendar time is set such that $t = 0$ corresponds to 90 days prior to the most recent observation. Parameter k_z controls the smoothness of logistic function. We set $k_z = 0.01$, resulting in a range of weights between $0.06 - 0.7$ for the duration of observation period.

We set $w_h = 0.89$, $w_u = 0.01$, and $w_d = 0.1$. We place a large weight on the hospitalizations census, since this time

medRxiv preprint doi: https://doi.org/10.1101/2020.06.12.20126391; this version posted April 23, 2021. The copyright holder for this preprint (which was not certified by peer review) is the author/funder, who has granted medRxiv a license to display the preprint in perpetuity.
It is made available under a CC-BY 4.0 International license .

series is most sensitive to changes in epidemic dynamics, and a small weight on cumulative hospitalizations, since it measures a feature that is related to hospitalizations census. The range of observation times differ for different time series. For hospitalizations census and deaths, observation times start with the first non-zero observation. For cumulative hospitalizations, observation times start on May 29, 2020 when these data started being reported routinely.

Posterior sampling

Sampling from the joint posterior distribution of $(\boldsymbol{\theta}, \boldsymbol{\sigma})$ given in (5) is performed using Markov Chain Monte Carlo (MCMC). We employ a hybrid algorithm that combines elliptical slice sampling (ESS) [59], Gibbs sampling, and Metropolis-Hastings sampling with random walk proposals. We first provide an overview of the steps in drawing samples from the full posterior distribution and then describe each steps in more details. Let $\boldsymbol{\theta} = (\boldsymbol{\theta}_{\text{MH}}, \boldsymbol{\theta}_{\text{ESS}})$, where $\boldsymbol{\theta}_{\text{MH}} = (\beta_0, q_A, \alpha_{I_S}, m_{H,0}, E_0, L_H, L_D)$ and $\boldsymbol{\theta}_{\text{ESS}} = (\tau, \boldsymbol{\epsilon})$. The sampler proceeds with the following steps:

1. Update $\boldsymbol{\theta}_{\text{ESS}} | \boldsymbol{\theta}_{\text{MH}}, \boldsymbol{\sigma}$ using ESS.
2. Update $\boldsymbol{\theta}_{\text{MH}} | \boldsymbol{\theta}_{\text{ESS}}, \boldsymbol{\sigma}$ with a Metropolis-Hastings step.
3. Update hyperparameters $\boldsymbol{\sigma} | \boldsymbol{\theta}$ with a Gibbs sampler step.

Update $\boldsymbol{\theta}_{\text{ESS}} | \boldsymbol{\theta}_{\text{MH}}, \boldsymbol{\sigma}$: We use a rejection-free sampler (ESS) to sample a vector of random effects $\boldsymbol{\epsilon}$ and a testing effect τ. The ESS operates by drawing samples from the ellipse defined by a Gaussian prior, and then accept or reject the samples by evaluating the likelihood component. Within the slice sampling step, the sampler moves along the generated ellipse and always accepts a new set of parameters.

Update $\boldsymbol{\theta}_{\text{MH}} | \boldsymbol{\theta}_{\text{ESS}}, \boldsymbol{\sigma}$: We implement a Metropolis-Hastings algorithm with random walk proposals for $\boldsymbol{\theta}_{\text{MH}}$. Proposals for L_H and L_D are made on a subset of integers bounded by a prior distribution on lags. All other elements of $\boldsymbol{\theta}_{\text{MH}}$ are continuous.

Update hyperparameters $\boldsymbol{\sigma} | \boldsymbol{\theta}$: The hyperparameters σ_h^2, σ_u^2, σ_d^2, σ_ϵ^2 are updated with Gibbs sampler steps:

$$\frac{1}{\sigma_h^2} | h(t), H(t) \sim \text{Gamma}\left(a + \frac{w_h \sum_{t \in t_H} z(t)}{2}, b + \frac{w_h \sum_{t \in t_H} z(t)(h(t) - H(t))^2}{2}\right)$$

medRxiv preprint doi: https://doi.org/10.1101/2020.06.12.20126391; this version posted April 23, 2021. The copyright holder for this preprint (which was not certified by peer review) is the author/funder, who has granted medRxiv a license to display the preprint in perpetuity.
It is made available under a CC-BY 4.0 International license .

$$\frac{1}{\sigma_u^2}|u(t), U(t) \sim \text{Gamma}\left(a + \frac{w_u \sum_{t \in t_U} z(t)}{2}, b + \frac{w_u \sum_{t \in t_U} z(t)(u(t) - U(t))^2}{2}\right)$$

$$\frac{1}{\sigma_d^2}|d(t), D(t) \sim \text{Gamma}\left(a + \frac{w_d \sum_{t \in t_D} z(t)}{2}, b + \frac{w_d \sum_{t \in t_D} z(t)(d(t) - D(t))^2}{2}\right)$$

$$\frac{1}{\sigma_\epsilon^2}|\epsilon_1, ..., \epsilon_K \sim \text{Gamma}\left(a_\epsilon + \frac{K}{2}, b_\epsilon + \frac{\sum_{i=1}^K (\epsilon_i - \epsilon_{i-1})^2}{2}\right)$$

We ran 10 chains of the sampler for 22,000 iterations each, discarded the first 2,000 draws from each chain, and thinned each chain by a factor of 20. The final posterior sample combines the resulting thinned chains. Based on the visual inspection of individual parameter trace plots, we found that 10,000 iterations is sufficient for the chain to converge in practice. Figure 6 illustrates model calibration results and convergence. The top row shows each of 10 thinned MCMC sampler chains overlaid. The middle row shows posterior histograms, marginal means and 95% posterior credible intervals of calibrated model parameters. The bottom row shows calibrated random effects function $B(t)$. To generate uncertainty intervals of model projections, we sample from the joint posterior over estimated parameters, and find pointwise 90% or 95% posterior predictive intervals for each time point.

Prior and posterior distributions of model parameters

Let $\boldsymbol{\theta}_{\text{MH,CONT}}$ denote the subset of continuous transmission model parameters whose joint distribution is calibrated to observed data and updated with Metropolis-Hastings step: $\boldsymbol{\theta}_{\text{MH, CONT}} = (\beta_0, q_A, \alpha_{I_S}, m_{H,0}, E_0)$. For each individual parameter $\theta \in \boldsymbol{\theta}_{\text{MH,CONT}}$, we specify a fixed support $[\theta_{\min}, \theta_{\max}]$, and put independent beta priors on the transformed parameter, i.e.,

$$\frac{\theta - \theta_{\min}}{\theta_{\max} - \theta_{\min}} \sim \text{Beta}(a_\theta, b_\theta), \quad (7)$$

where the shape parameters a_θ and b_θ are set to let θ have mean μ_θ and standard deviation σ_θ. Table 2 provides the summary of $(\mu_\theta, \sigma_\theta, \theta_{\min}, \theta_{\max})$ for all parameters in $\boldsymbol{\theta}_{\text{MH,CONT}}$ along with data sources. For parameters whose values were fixed, only the mean is given. Parameter τ is updated in the ESS step, therefore in calibration, we assume a Gaussian prior distribution on $\log(\tau)$, whose mean and SD are shown in Table 2.

Asymptomatic infections play an important role in transmission of SARS-CoV-2 [85–87], but estimates of the proportion of infections that do not exhibit symptoms vary substantially [60, 61, 88]. The true proportion of asymptomatic infections is important for projections and policy planning due to its relationship to evolving herd

medRxiv preprint doi: https://doi.org/10.1101/2020.06.12.20126391; this version posted April 23, 2021. The copyright holder for this preprint (which was not certified by peer review) is the author/funder, who has granted medRxiv a license to display the preprint in perpetuity.
It is made available under a CC-BY 4.0 International license .

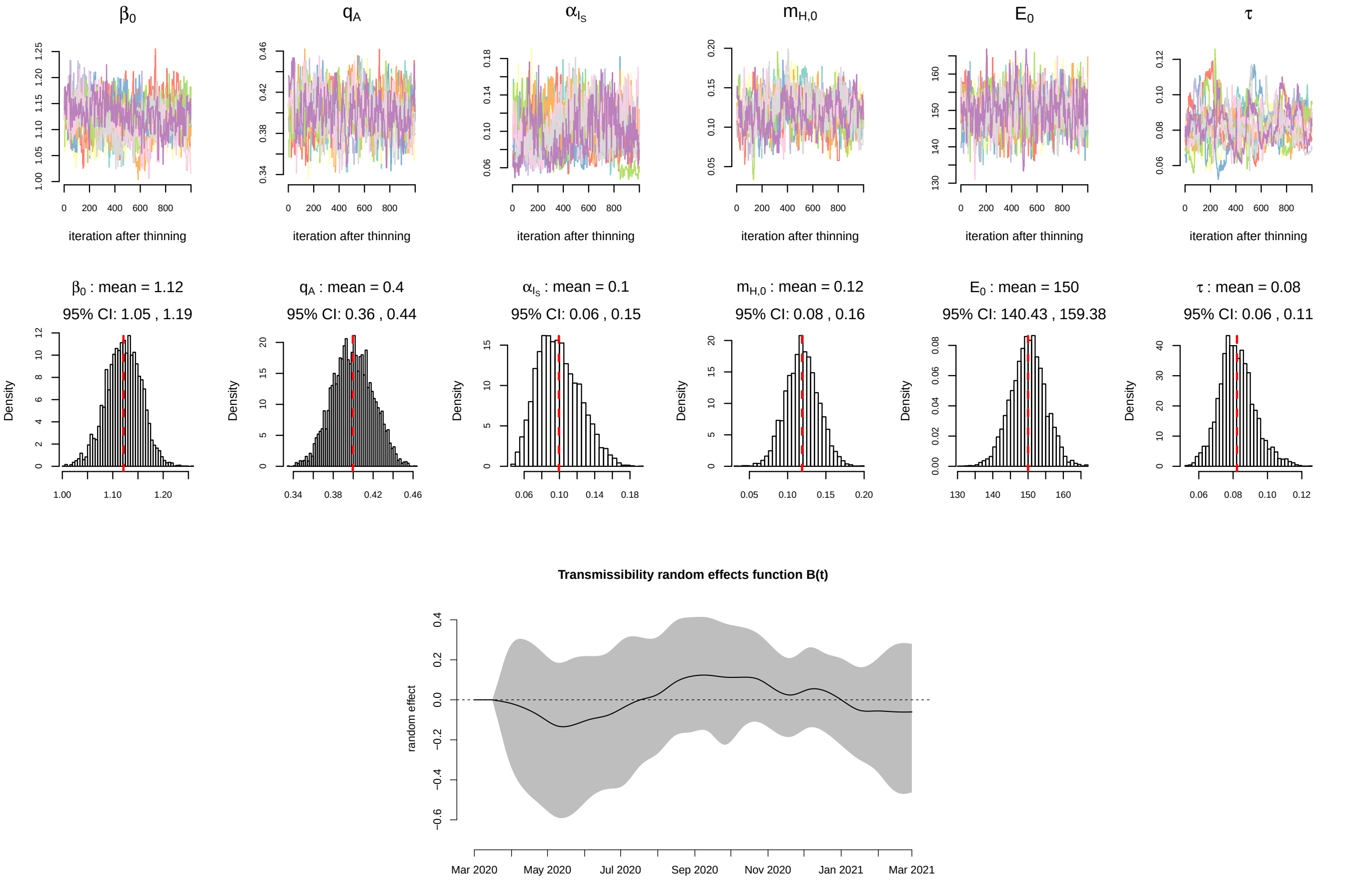

Figure 6: Model calibration results: trace plots of MCMC sampler chains, marginal posterior histograms of calibrated parameters, and random effects function $B(t)$. MCMC trace plots show 10 thinned chains plotted using different colors. Plot titles of posterior histograms include marginal posterior means and 95% credible intervals (CI). Dashed red lines correspond to posterior means of model parameters. In the transmissibility random effects function plot, black line shows the mean value of $B(t)$ at each time point, and the shaded region shows 95% CI.

medRxiv preprint doi: https://doi.org/10.1101/2020.06.12.20126391; this version posted April 23, 2021. The copyright holder for this preprint
(which was not certified by peer review) is the author/funder, who has granted medRxiv a license to display the preprint in perpetuity.
It is made available under a CC-BY 4.0 International license .

Table 2: Prior distributions of model parameters.

Parameter	Mean	SD	Lower	Upper	Source
q_A	0.4	0.02	0.3	0.5	[60–65]
$q_{I_S,0}/(1-q_A)$	0.07	-	-	-	7% of symptomatic infections are severe [4, 6, 49]
β_0	1	0.5	0.01	2.00	A diffuse prior assumed
δ	1/4	-	-	-	[6, 12, 15, 23, 62, 66–73]
$\alpha_{A,0}$	1/7	-	-	-	[66, 74–79]
k_A	0.5	-	-	-	[23, 61, 76, 80, 81]
$\alpha_{I_M,0}$	1/4	-	-	-	[4, 6, 15]; of 4 days, 2 are assumed to be presymptomatic [23, 66, 67]
γ_{R_M}	1/7	-	-	-	[74, 78, 79, 82]
α_{I_S}	1/9	0.03	0.02	0.2	[23, 66, 67, 83, 84]
$m_{H,0}$	0.13	0.05	0.01	0.25	Estimated from Connecticut COVID-19 hospitalization data [41]
$m_{\bar{H}}/m_H$	1.5	0.25	1	2	Assumed
$\log(\tau)$	-2.5	0.12	-	-	Assumed, Gaussian prior distribution
k_n	0.015	-	-	-	Assumed
E_0	150	5	100	200	Assumed
L_H	-	-	5	14	Assumed, uniform prior between lower and upper values
L_D	-	-	5	14	Assumed, uniform prior between lower and upper values

immunity. Reported estimates of asymptomatic proportion range between 6 – 96%, and the authors of a recent review recommend a range between 40 – 45% [60]. Another review reported an overall asymptomatic proportion estimate of 20%, and 31% among the studies that included follow-up [61]. Large population-based studies conducted in Spain and in the U.K. provide estimates of asymptomatic proportion between 22-36% [89, 90]. We calculated age-adjusted weighted average of several estimates available from the literature: Nishiura et al. [63] estimated 30.8% among Japanese citizens evacuated from Wuhan, China. We applied this estimate to age group 20-64 years old. Mizumoto et al. [64] estimated 17.9% among infections on the Diamond Princess cruise ship. We applied this estimate to age group 65 plus years old, which is the age group, in which most infections occurred. We assumed 65% for age group 0-19 years old, consistent with findings reported by Russell et al. [65], where 4 out of 6 infections in this age group were asymptomatic among passengers of the Diamond Princess. The average was weighted by the age distribution of Connecticut population, resulting in the estimate of $q_A = 0.37$, which is consistent with systematic reviews and population-based studies. At the time of this writing, the best estimate of asymptomatic proportion recommended by the CDC was 40% [62]. We center the prior distribution of q_A at 0.4 and allow it to vary between 0.3 and 0.5. The proportion of severe cases early in the epidemic ($q_{I_S,0}$) is assumed to be 7% of symptomatic infections in line

medRxiv preprint doi: https://doi.org/10.1101/2020.06.12.20126391; this version posted April 23, 2021. The copyright holder for this preprint (which was not certified by peer review) is the author/funder, who has granted medRxiv a license to display the preprint in perpetuity.
It is made available under a CC-BY 4.0 International license .

with [4, 6, 49]. Combined with the estimates of asymptomatic proportion, this translates into an estimate of severe proportion of 4.2% (3.5-4.9%) of all infections.

Duration of latency ($1/\delta$) was fixed, since consistent estimates of this parameter are available from the literature, and because it is highly correlated with several calibrated parameters, including reporting lags and parameters that determine duration of infectiousness and time between infection and recovery or death. We assume an average of 4 days of latency [6, 15, 23] and 2 days of presymptomatic infectiousness [23, 66, 67] resulting in an average incubation period of 6 days consistent with [12, 62, 68–73].

Parameters (α_A, k_A, α_{I_M}, α_{I_S}) collectively determine the force of infection at any given time. Force of infection, transmission parameter β and initial number of exposed individuals E_0 together determine the early growth of the epidemic. Without additional data, all of these parameters cannot be simultaneously identified. We therefore fixed the values for a subset of these parameters based on available estimates and assumed a diffuse prior on β, which absorbs additional variation of parameters that determine the force of infection.

Duration of infectiousness of asymptomatic individuals is unknown, but is likely shorter than that of symptomatic individuals [74–77]. While several studies estimated that viral RNA could be detected in upper respiratory tract for 2-3 weeks [91, 92], findings from [74, 78, 79] show that in mild-to-moderate symptomatic patients live virus could be isolated for a substantially shorter time period: up to 7-10 days from the day of symptom onset, suggesting the duration of infectiousness of up to 12 days. Based on these estimates and [66], we assume the duration of infectiousness of 7 days among asymptomatic individuals. Although multiple studies have shown similar viral loads among symptomatic, presymptomatic and asymptomatic cases [75, 93, 94], evidence suggests that asymptomatic individuals are less infectious that symptomatic, likely due to higher viral shedding while coughing and longer duration of infectiousness among symptomatic individuals [61, 74, 76, 95, 96]. Multiple estimates of relative infectiousness of asymptomatic individuals compared to symptomatic are available and range from close to zero to above one [23, 61, 76, 80, 81]. Generally, estimates that are based on attack rates are lower than those based on viral shedding or time until the first negative PCR test. In our analysis, we set relative infectiousness of asymptomatic cases to be 0.5 [23, 61, 80].

Although the duration of infectiousness of mild-to-moderately symptomatic cases may be up to 12 days [74, 78, 79], we assume that the majority of symptomatic individuals self-isolate shortly after developing symptoms. We set the duration of infectiousness of symptomatic cases to be 4 days [4, 6, 15], 2 of which are assumed to represent presymptomatic infectiousness [23, 66, 67]. We further assume that the duration of self-isolation until recovery is 7

medRxiv preprint doi: https://doi.org/10.1101/2020.06.12.20126391; this version posted April 23, 2021. The copyright holder for this preprint (which was not certified by peer review) is the author/funder, who has granted medRxiv a license to display the preprint in perpetuity.
It is made available under a CC-BY 4.0 International license .

days [74, 78, 79].

We calibrate the rate of hospitalization among severe cases (α_{I_S}), assuming a mean of 9 days between the onset of infectiousness and hospitalization: 2 days of presymptomatic infectiousness plus 7 days between the onset of symptoms and hospitalization [83, 84].
